# Effectiveness and Safety of Low-Dose Propofol for the Treatment of Migraine in the Emergency Department: A Matched Retrospective Cohort Study

**DOI:** 10.7759/cureus.88930

**Published:** 2025-07-28

**Authors:** Stéphane Côté, David Simonyan, Myriam Mallet, Simon Baril, Laurie Ouellet, Simon Berthelot

**Affiliations:** 1 Department of Emergency Medicine, Centre Hospitalier Universitaire (CHU) de Québec-Université Laval, Quebec, CAN; 2 Clinical and Evaluative Research Platform, Centre Hospitalier Universitaire (CHU) de Québec-Université Laval, Quebec, CAN; 3 Data Valorization and Exploitation Center, Centre Hospitalier Universitaire (CHU) de Québec-Université Laval, Quebec, CAN

**Keywords:** emergency department, headache, metoclopramide, migraine, propofol

## Abstract

Introduction

This study aimed to evaluate the effectiveness and safety of propofol compared to metoclopramide for the treatment of migraines in the emergency department (ED).

Methodology

We reviewed the health records of patients aged 16 years and older treated with propofol for migraine between 2014 and 2017 at a teaching hospital in Québec City. The local protocol consisted of administering propofol (20 mg) every 10 minutes as needed (up to six doses), vital signs before and after each dose, and continuous cardiac monitoring. The primary outcome measure was the mean reduction of pain following first-line therapy (propofol or metoclopramide). The secondary outcome measures were: (1) adjusted relative risks of requiring rescue medication after first-line therapy; and (2) the incidence of the following side effects associated with propofol administered as first- or second-line therapy: hypotension (systolic <90 mmHg or mean <65 mmHg), desaturation, excessive sedation, and arrhythmia. The cohorts were paired for gender, age, triage priority, and date of ED visit.

Results

Medical records of 34 patients treated with propofol and 58 patients treated with metoclopramide as first-line therapy were analyzed. Five patients in the metoclopramide group received propofol as rescue medication. Among those treated with propofol, 10 patients (29.4%) experienced pain relief, compared to 38 (66%) in the metoclopramide group (*P* < 0.001). Rescue medication was more frequent in first-line propofol patients (28 patients, 82.4%, versus 22, 37.9%, *P* < 0.001). In this group, four participants (10.3%) received an intravenous fluid bolus for a mean blood pressure below 60 mmHg, but no other persistent adverse effects were recorded.

Conclusions

Though less effective than metoclopramide, propofol at low doses may be an alternative to treat migraine in the ED. Monitoring of vital signs would be prudent, but continuous nursing is likely unnecessary.

## Introduction

Migraine is a common cause of emergency department (ED) visits [1]. Current best estimates indicate that the global prevalence of migraine is 14%-15% and that it accounts for 4.9% of all years lived with disability worldwide [2]. Migraine patients presenting to the ED have often experienced therapeutic failure [3], and identifying a safe and effective pharmacological option for rapid relief remains challenging.

The targets of medication are often multiple and complex, and numerous options have been used to treat acute migraine. The most common of these include nonsteroidal anti-inflammatory agents, neuroleptics, dopamine-antagonists (e.g., metoclopramide), and triptans [4]. Subanesthetic doses of propofol have been proposed as a potential alternative, although the evidence remains limited and variable across studies [5-12]. Its mechanism of action in migraine is not well established, but it likely involves gamma-aminobutyric acid type A (GABAa) receptor agonism, producing sedative and anxiolytic effects [5,13]. GABA receptor agonism affects calcium channels and may also inhibit N-methyl-D-aspartate (NMDA) receptors, leading to neuronal hyperpolarization and inhibition of the neuronal firing that might lead to migraine, though this remains hypothetical [5,13]. One of the challenges of using propofol is its significant side-effect profile, which includes arterial hypotension and respiratory depression [14,15]. Although low doses are used to treat migraine, the patients need more monitoring than with other alternatives, since the safety of propofol remains to be demonstrated unequivocally for this indication.

This study aimed to (1) compare the effectiveness (i.e., pain reduction and use of rescue medication) of propofol with that of metoclopramide and (2) assess its safety (i.e., side effects) for the treatment of migraines in the ED.

This article was previously posted to the medrXiv preprint server on October 10, 2024; DOI: 10.1101/2024.10.09.24315176.

## Materials and methods

Study design and setting

We conducted a health records review of patients who received intravenous propofol as a first-line or second-line ED treatment for migraine at the Centre Hospitalier de l'Université Laval (CHUL, a teaching hospital in Quebec City, Canada, receiving 78,000 visits annually) between May 2014 and August 2017. Patients were identified through a mandatory specific prescription form that emergency physicians had to fill when using propofol for this off-label indication. For comparison purposes, we also extracted clinical data from a cohort of patients with migraine who received metoclopramide initially. The two cohorts were paired for sex, age, triage priority, and month/year of the ED visit. This study received approval from the CHUL research ethics board (authorization no. 2017-3012).

Participants

We reviewed and included all charts of patients who received propofol during the study period if they were at least 16 years old and treated for migraine without aura or with typical aura as defined in the International Headache Society classification [16]. Patients treated with propofol for headaches not meeting the International Headache Society diagnostic criteria and those with contraindications identified in the local care protocol were excluded from the analysis. These contraindications included pregnancy, severe coronary artery disease (American Society of Anesthesiologists (ASA) ≥ 3), heart failure, severe chronic pulmonary disease (FEV1 < 50%), familial dyslipidemia, neuromuscular disease, oxygen dependence, hemodynamic instability, and known allergy to egg or soy. Since we planned to include all eligible patients with migraine who received propofol, no sample size calculation was performed.

Therapeutic guide

A therapeutic guide implemented in May 2014 for using propofol to treat migraine specifies administering 20 mg (intravenously) every 5 to 10 minutes as needed, up to six doses (Appendix). Vital signs and pain assessment are recorded before and after each dose. Cardiac and saturation monitoring is continuous. A bedside nurse assesses the patient’s pain score, state of consciousness and hemodynamic parameters throughout administration of the drug. Patients are considered relieved, and administration is stopped, when the pain score decreases to 2 or less on a scale from 0 (no pain) to 10 (worst pain).

Data sources and collection

All patient electronic records were extracted by two independent evaluators, and disagreements with the charts were resolved by discussion. A third evaluator participated when the discussion did not reach a consensus. Demographic and clinical characteristics were collected on a standardized Excel spreadsheet and included the following variables: age; biological sex; time, date and month of ED visit; comorbidities; regular medication; vital signs; pain assessment (10-level) and sedation on the four-level Pasero scale [17], from 1 (easy to rouse) to 4 (unresponsive) on ED arrival and discharge, pre- and post-administration of propofol; use of co-analgesics or rescue medication. The Pasero scale is used in the participating ED to monitor sedation in patients who receive sedative medications, such as opioids. Since metoclopramide administration was not standardized in a protocol, pain assessment was not translated systematically to the 10-level scale when the patient was discharged. When no final pain score was recorded on the chart of a patient discharged after treatment with metoclopramide, we extrapolated a score from the nurse's discharge note, assigning a score of 2 if partial or almost complete pain relief was mentioned, and 0 if the nurse specifically indicated that the patient had no pain at discharge.

Outcomes

The primary outcome measure was the mean reduction of pain intensity after first-line treatment (propofol or metoclopramide). The secondary outcomes measures were (1) adjusted relative risk of requiring rescue medication; (2) incidence of the following side effects for patients who received propofol (as first-line therapy or rescue medication): low arterial pressure (<90 systolic mmHg or <65 mmHg mean), desaturation (SaO2 < 92%), excessive sedation (Pasero score of 3 or 4), and any arrhythmia.

Statistical analysis

Patient characteristics are presented as means, counts, and proportions. Mean pain scores pre- and post-propofol (first- and second-line treatments) were compared using a generalized linear regression model (estimating equations) designed for repeated measures. Incidences of adverse effects are reported as proportions with 95% confidence intervals. We also compared propofol to metoclopramide using student’s t-test on the mean differences of pre- and post-treatment pain scores. A chi-square test was conducted to compare the proportions of each group that needed rescue medication. The adjusted relative risk was estimated for this need using a log-binomial regression model and a propensity score generated using a logistic regression model with treatment groups (propofol versus metoclopramide) as the dependent variable. The independent variables were age, sex, anti-migraine medication taken before arriving at the ED, anti-migraine medication administered concomitantly with propofol or metoclopramide, triage score, chronic obstructive pulmonary disease, chronic use of antihypertensives or anxiolytics, and clinical parameters upon arrival in the ED, including pain level, oxygen saturation, and systolic and diastolic blood pressure. All analyses were performed with SAS version 9.4 (SAS Institute, Cary, NC), with a two-sided significance level set at *P* < 0.05.

## Results

The demographics and other basic characteristics of the participants are summarized in Table 1. Over the three-year study period, 34 participants received propofol as first-line treatment in accordance with the therapeutic guide. All patients who received propofol for migraine during the study period met the inclusion criteria. After assessing the eligibility of 92 migraine cases matched to the propofol cases based on age, sex, triage priority, and month/year of ED visit, 58 patients who had received metoclopramide as first-line treatment were retained for the comparative analysis. Among these, 5 received propofol as rescue medication. They are described separately in Table 1.

**Table 1:** Characteristics of the participants. ^a^All data are reported as *n *(%), unless otherwise indicated. ^b^All patients who received propofol as second-line medication received metoclopramide as their first antimigraine agent in the ED and are, therefore, included in the metoclopramide group. ^c^Standard deviation. ^d^Given at the same time as propofol and metoclopramide.

Characteristic^a^	Propofol first line (*n* = 34)	Propofol second line^b^ (*n* = 5)	Metoclopramide (*n* = 53)
Mean age in years (SD)^c^	37.1 (14.7)	45.6 (14.7)	34.8 (12.4)
Female, *n* (%)	27 (79.4)	4 (80.0)	43 (81.1)
Canadian Triage and Acuity Score			
2	6 (17.6)	1 (20.0)	9 (17.0)
3	24 (70.6)	4 (80.0)	41 (77.3)
4	4 (11.8)	0 (0)	3 (5.7)
Mean vital signs at triage (SD)			
Systolic blood pressure (mmHg)	130.3 (17.3)	126.6 (17.6)	132.1 (19.0)
Diastolic blood pressure (mmHg)	82.2 (11.8)	83.8 (8.0)	83.7 (10.8)
Oxygen saturation (%)	99.1 (1.3)	99.3 (1.5)	98.7 (1.5)
Respiratory rate (breaths/minute)	18.6 (4.7)	17 (1.4)	17.7 (2.6)
Comorbidities			
Hypertension	2 (5.9)	0 (0)	6 (11.3)
Coronary artery disease	0 (0)	0 (0)	2 (3.8)
Asthma/Chronic pulmonary disease	3 (8.8)	1 (20.0)	10 (18.9)
Regular medication			
Opioids	0 (0)	0 (0)	2 (3.8)
Benzodiazepines	0 (0)	1 (20.0)	3 (5.7)
Antihypertensives	4 (11.8)	0 (0)	5 (9.4)
Antimigraine taken prior to arrival at the ED	22 (64.7)	3 (60.0)	41 (77.4)
Received concomitant medication, *n* (%)^ d^	4 (11.8)	4 (80.0)	43 (81.1)
Acetaminophen	1 (2.9)	1 (20.0)	14 (26.4)
Naproxen	2 (5.9)	2 (40.0)	16 (30.2)
Dexamethasone	2 (5.9)	1 (20.0)	5 (9.4)
Lorazepam	0 (0)	1 (20.0)	4 (7.5)
Diphenhydramine	0 (0)	1 (20.0	21 (39.6)
Opioids	0 (0)	0 (0)	0 (0)

There were no significant differences in age, sex, or comorbidity between the propofol and metoclopramide first-line groups. The baseline vital signs and pain scores at ED triage were also comparable. However, 7 (12.7%) more participants in the metoclopramide group had taken anti-migraine medication before arriving at the ED.

**Figure 1:**
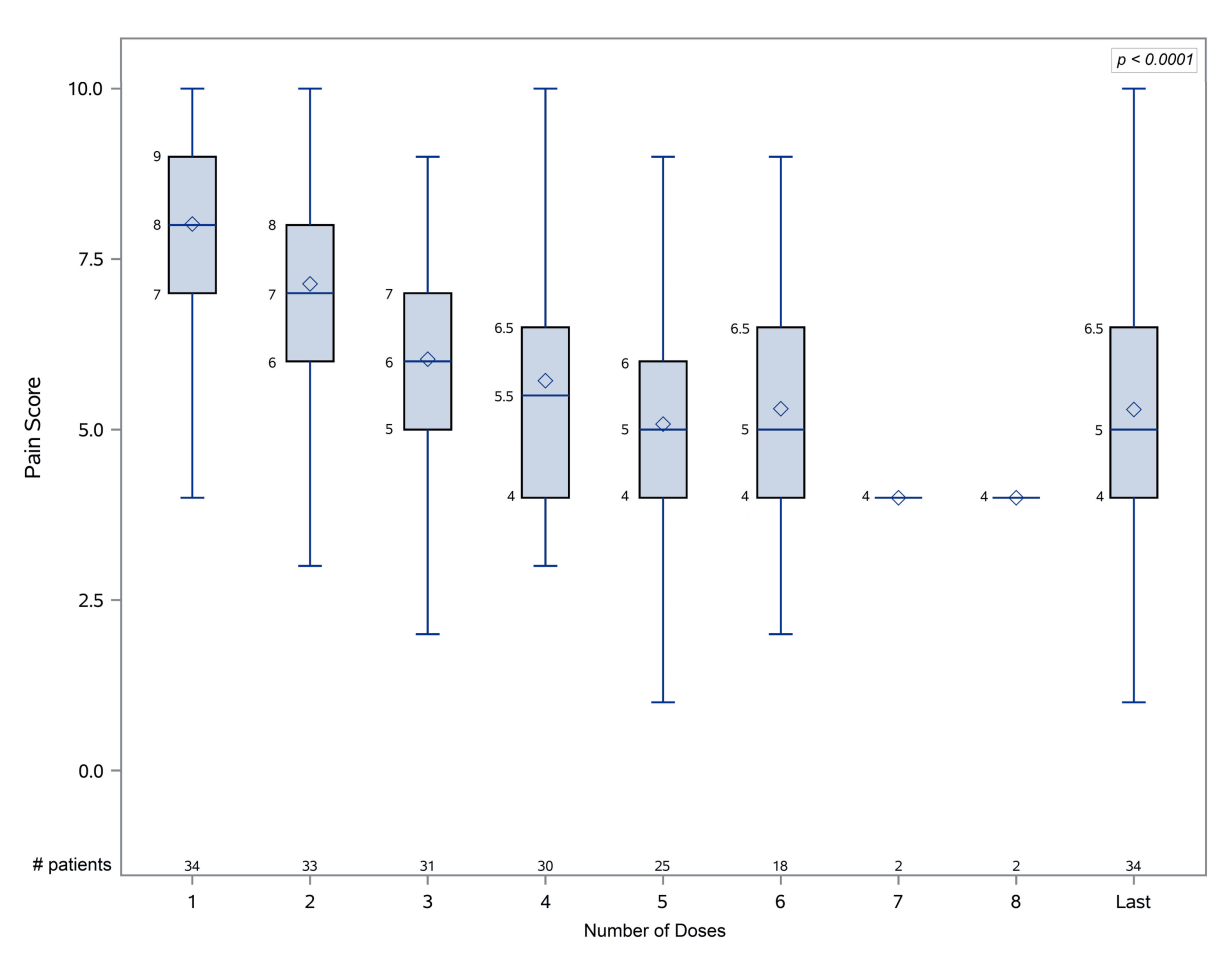
Box plot of migraine pain intensity recorded following repeated low doses of propofol as a first-line therapy (n = 34). # patients = number of patients included in the analysis for each dose. The *P*-value represents the comparison between the first and last doses for all patients.

As shown in Table 2 and Figure 1, repeated doses of propofol (first or second line) decreased pain intensity significantly up to the sixth dose. However, only 10 (29.4%) patients given first-line treatment with propofol achieved a pain level of 2 or less, compared to 38 (66%) patients given metoclopramide who had comparable pain levels upon arrival in the ED (*P* < 0.001). However, 47 (81.1%) patients received co-analgesic medications, compared to only 4 (11.8%) patients treated with propofol.

**Table 2:** Mean pain score recorded before and after first-line treatment with propofol or metoclopramide a Based on Student’s t-test b From 0 (no pain) to 10 (worst pain) c All results are reported as means with 95% confidence intervals, unless otherwise indicated

Measurement	Propofol (n = 34)	Metoclopramide (n = 58)	P value ^a^
Initial pain score ^b^	8.2 (7.6-8.7)^c^	7.7 (7.2-8.2)	0.21
Final pain score	4.3 (3.3-5.2)	2.1 (1.3-2.9)	< 0.001
Before-after difference	3.8 (2.9-4.8)	5.6 (4.8-6.4)	0.006
Length of ED stay (hours)	7.0 (5.2-8.9)	7.5 (5.2-9.8)	0.78
Patients n (%) relieved of pain (score ≤ 2)	10 (29.4)	38 (66)	< 0.001

The proportion of patients who required a rescue medication was higher in the propofol group (28, 82.4%, vs. 22, 37.9%, *P* < 0.001; Table 3). The adjusted risk of requiring rescue medication was 81% higher for this group. In 23 (67%) of rescues in the propofol group, the medication used was metoclopramide, whereas propofol was used in only 5 (8.6%) of the metoclopramide group needing rescue.

**Table 3:** Rescue medications used after ED treatment of migraine with propofol (n = 34) or metoclopramide (n = 58). ^a^Meaning any drug administered after the initial migraine medication, to treat persistent pain or an adverse reaction (e.g., nausea). ^b^Relative risk of requiring rescue, adjusted using a propensity score including age, sex, anti-migraine medication taken before arriving at the ED, triage score, chronic obstructive pulmonary disease, chronic use of antihypertensives or anxiolytics, and clinical parameters upon arrival in the ED, including pain level, oxygen saturation, and systolic and diastolic blood pressure. (aRR > 1 means rescue medication used more after treatment with propofol; aRR < 1 means rescue medication used more after treatment with metoclopramide.) ^c^Confidence interval of aRR. aRR, adjusted risk ratio

Medication^a^	Propofol, *n* (%)	Metoclopramide, *n* (%)	*P*-value	aRR^b^	95% CI^c^
Acetaminophen	6 (17.6)	6 (10.5)	0.35	1.39	0.46-4.16
Naproxen	11 (32.4)	6 (10.3)	0.01	2.89	1.09-7.62
Opioids	2 (5.9)	7 (12.1)	0.48	0.47	0.08-2.65
Dexamethasone	8 (23.5)	9 (15.5)	0.41	1.21	0.42-3.49
Lorazepam	3 (8.8)	2 (3.4)	0.35	3.37	0.69-16.49
Dimenhydrinate	0	4 (7.0)	0.29	-	-
Diphenhydramine	0	1 (1.7)	1.0	-	-
Ondansetron	0	1 (1.7)	1.0	-	-
Sumatriptan	0	3 (5.2)	0.29	-	-
Haloperidol	0	1 (1.7)	1.0	-	-
Dihydroergotamine	0	1 (1.7)	1.0	-	-
Metoclopramide	23 (67.6)	0	<0.001	-	-
Propofol	0	5 (8.6)	0.15	-	-
Total	28 (82.4)	22 (37.9)	<0.001	1.81	1.23-2.68

Among recipients of propofol as a first-line treatment (*n* = 34) or as a rescue medication (*n* = 5), no significant or persistent desaturation (SaO2 < 92%), bradycardia (heart rate < 60 bpm) or excessive sedation (Pasero score 3 or 4) were recorded. At the end of first-line treatment, 19 (54.5%) participants had a transient decrease in their mean blood pressure to below 60 mmHg and 2 (6.1%) had a systolic blood pressure below 90 mmHg. Intravenous fluid bolus was undertaken for four participants (10.3%) in the propofol group, but no vasopressor was needed.

## Discussion

Interpretation of findings

This review of health records shows that repeated low doses of propofol are effective at reducing pain in patients presenting with migraines. No desaturation or decreased level of consciousness occurred, although 4 patients (10%) received a fluid bolus for minor drops in blood pressure. However, metoclopramide appears to be more effective in the ED setting: more patients felt their pain reduced to 2 or lower, and fewer required rescue medication. Nevertheless, propofol administered at low doses appears to be a potential alternative for this indication, requiring only blood pressure monitoring to ensure patient safety.

Comparison to previous studies

The ED use of propofol to treat migraines has been reviewed systematically twice in recent years. The first of these [18] considered 9 studies that covered 290 patients who received propofol as first-line treatment: five case reports or case series, one retrospective cohort study, and three randomized controlled studies. The subsequent review [19] focused on the effectiveness of parenteral agents. We also examined four randomized trials [20-23] of propofol, including one with metoclopramide or granisetron as a co-intervention for nausea [24]. Despite the design problems of reviews based on health records, our study included a cohort of 92 patients, comparable in size to the largest randomized studies to date [20,24]. Although propofol was found not superior to placebo in a cohort study of 40 patients [23], all other reports indicate that this molecule is effective for the treatment of migraine. We found that 10 patients (29.4%) given propofol as first-line treatment were relieved completely of pain (score ≤ 2) and a mean pain reduction of 46.3% was obtained. This diverges from studies in which 84.4% of the participants were relieved of pain (20) or the mean migraine pain score was reduced by 87.5% [24]. The lower effectiveness observed in our study may be due to differences in the cohorts and protocols. Only 22% of our propofol group had received medication before admission to the ED, versus 93.3% [20]. Furthermore, we administered propofol in intravenous doses of 20 mg for a total of 120 mg, versus an initial 30-40 mg subcutaneous dose followed by boluses of 10-20 mg up to a total of 120 mg. Finally, we considered that the patient was relieved of pain when the score reached 2 or less on the 10-level scale, versus defining responsiveness to therapy as a reduction of at least 4 points on the visual analog scale.

The other point to consider when using propofol is its safety. The adverse effects of propofol (cardiovascular, e.g., bradycardia, hypotension; respiratory, e.g., ventilatory depressant, desaturation, hypoxemia, apnea) are well documented and controlled with constant monitoring by qualified medical personnel [14,15]. Since typical ED staff regard with apprehension the use of propofol outside intensive care units or resuscitation rooms, we implemented a strict protocol to evaluate side effects. Our results confirm previous reports that using propofol at subanesthetic doses causes very few side effects [18,19]. The safety profile of low-dose propofol in the ED for treatment of migraine thus appears favorable.

Strengths and limitations

A study based on the review of health records has inherent limitations that should be considered when interpreting the results. One major limitation is the retrospective and non-randomized design, which may introduce selection bias and unmeasured confounding factors. Patients were not randomized to receive either propofol or metoclopramide; rather, the choice of treatment was based on clinical judgment, which may reflect differences in patient characteristics or provider preferences. Consequently, observed differences in outcomes might not be solely attributable to the pharmacological effect of the drugs.

Differences in baseline pain scores between groups, particularly higher initial pain intensity in the propofol group, could also have influenced treatment outcomes. To address this potential confounder, we included baseline pain scores as a covariate in both our multivariable regression and propensity score analyses. This analytic strategy allowed us to adjust for initial differences in pain severity and better isolate the treatment effect. While residual confounding cannot be entirely ruled out, we believe this approach adequately addresses the concern.

Another limitation is the use of data collected between 2014 and 2017. While we acknowledge that clinical practices, particularly in migraine management, may evolve over time, our primary objective was to assess the effectiveness and safety of low-dose propofol-a treatment that remains off-label and is still not widely adopted in emergency departments. The direct comparison with metoclopramide, which continues to be a commonly used agent, and the real-world nature of the data, strengthen the relevance of our findings. Furthermore, the rigorous monitoring protocol used in the propofol group provides valuable safety data. Nonetheless, we recognize the importance of future prospective studies using contemporary data to confirm and expand upon our results.

Additionally, in the metoclopramide group, discharge pain scores were not consistently documented in the medical records, and we had to infer a score of 2 or less based on physician and nurse notes. While we cannot provide an exact count of how many patients were classified in this way, each case was reviewed independently by two evaluators, with discrepancies resolved through discussion. Importantly, the use of rescue medication - an objective secondary outcome not subject to this limitation - provides further support for the superiority of metoclopramide as a first-line treatment. Moreover, the greater use of co-analgesics in the metoclopramide group may have contributed to improved pain relief in this group, limiting the comparability of analgesic efficacy between treatments.

Finally, clinicians in the metoclopramide group were not required to follow a standardized protocol, and side effects such as extrapyramidal symptoms were not systematically recorded.

The main strength of our study lies in the structured propofol administration protocol, which ensured consistent data collection. Two robust indicators emerge: first, the need for rescue medication was objectively documented in both groups due to complete medication records; second, continuous monitoring in the propofol group supports our conclusion that no significant adverse events occurred.

Clinical implications

This study confirms previous findings that propofol may be considered a safe alternative for treatment of migraine in the ED. However, given the well-demonstrated effectiveness of metoclopramide, this agent may be the better first-line option. The care protocol prompting this study included very close monitoring of patients with nearly continuous nursing presence. The few side effects recorded demonstrate that propofol is safe, and at doses much lower than those used for procedural sedation or intubation, blood pressure monitoring is likely sufficient precaution. Our data suggest that propofol is safe enough to be used without constant bedside nursing.

Research implications

The effectiveness of propofol for the treatment of migraine is not negligible, but the scientific data available remain insufficient to justify its addition to the list of standard therapeutic options. Randomized controlled studies with larger sample sizes are needed to obtain a reliable estimation of its true effectiveness [25,26]. Furthermore, the doses of propofol currently used are chosen empirically and often differ from one study to another, which may explain its variable efficacy. A protocol must be developed to establish a pharmacokinetic dose-response curve (EC50). Patient weight-matched doses of propofol will help answer the questions that remain.

## Conclusions

In this study, we evaluated the effectiveness and safety of administering propofol to treat migraines in the ED. The results suggest that propofol can be a safe alternative as a second-line option after the administration of neuroleptics, primarily metoclopramide, which appears to be the first choice in many EDs. If propofol is administered, monitoring of vital signs, especially blood pressure, appears prudent, but continuous bedside nursing is likely unnecessary.

## Appendices

Appendix

Protocole d’administration du propofol dans la migraine

DOSSIER # _________   

ÂGE ____    POIDS : __________ kg     VAS initiale : ____   VAS finale : ____

Indications et contre-indications (verso)

PRÉPARATION

Installer le patient sous monitorage cardiaque (BLOC B OU C)

Prendre les signes vitaux (Pouls, TA, RR et SAO2)

Obtenir une voie veineuse et installer un soluté NaCl 0,9% en GVO

ADMINISTRATION

Ne pas administrer le Propofol si instabilité hémodynamique ou TA ≤ 90/50

Propofol 20 mg iv q 5-10 minutes (MAX: 120 mg) ou jusqu’à score VAS ≤ 2

Contrôle des paramètres vitaux 5 minutes après chaque dose

Surveiller l’apparition des effets secondaires suivants :

Hypotension

Dépression respiratoire

Allergie ou anaphylaxie

Évaluer la douleur sur l’échelle VAS, 5 minutes après chaque dose

Départ 30 minutes après la dernière dose si conscience et signes vitaux normaux

Indications

16 ans et plus

Migraine sans aura

Migraine avec aura typique (avec céphalée)

Migraine chronique   

      (céphalée ≥15 jours/mois avec les critères de migraine présents au moins 8 jours)                                               

Status migraineux

      (migraine présente depuis plus de 72 heures)

Contre-indications

Céphalée secondaire et autres céphalées primaires telles que définies par l’IHS

Allergie aux oeufs et soya et instabilité hémodynamique (TA) ≤ 90/50)

Grossesse

Comorbidité sévère

maladie cardiaque sévère (ASA ≥ classe III/IV)

Dyslipidémie familiale

maladie neuromusculaire

MPOC sévère et O2 dépendant
